# An improved YOLOv5s model using feature concatenation with attention mechanism for real-time fruit detection and counting

**DOI:** 10.3389/fpls.2023.1153505

**Published:** 2023-06-26

**Authors:** Olarewaju Mubashiru Lawal, Shengyan Zhu, Kui Cheng

**Affiliations:** Sanjiang Institute of Artificial Intelligence and Robotics, Yibin University, Sichuan, China

**Keywords:** improved YOLOv5s, fruit detection, fruit counting, feature concatenation, attention mechanism (AM)

## Abstract

An improved YOLOv5s model was proposed and validated on a new fruit dataset to solve the real-time detection task in a complex environment. With the incorporation of feature concatenation and an attention mechanism into the original YOLOv5s network, the improved YOLOv5s recorded 122 layers, 4.4 × 10^6^ params, 12.8 GFLOPs, and 8.8 MB weight size, which are 45.5%, 30.2%, 14.1%, and 31.3% smaller than the original YOLOv5s, respectively. Meanwhile, the obtained 93.4% of mAP tested on the valid set, 96.0% of mAP tested on the test set, and 74 fps of speed tested on videos using improved YOLOv5s is 0.6%, 0.5%, and 10.4% higher than the original YOLOv5s model, respectively. Using videos, the fruit tracking and counting tested on the improved YOLOv5s observed less missed and incorrect detections compared to the original YOLOv5s. Furthermore, the aggregated detection performance of improved YOLOv5s outperformed the network of GhostYOLOv5s, YOLOv4-tiny, and YOLOv7-tiny, including other mainstream YOLO variants. Therefore, the improved YOLOv5s is lightweight with reduced computation costs, can better generalize against complex conditions, and is applicable for real-time detection in fruit picking robots and low-power devices.

## 1 Introduction

The demand for fruit is increasing every day because it provides essential nutrients and several health benefits for humans (Pal and Molnar, 2021). Over 841 million metric tons of fruit was reported to have been produced in the year 2020 according to Shahbandeh (2022). However, in the current fruit demand and production status, there is a strict time limit for fruit picking and the demand for labor is large, which means that labor costs will increase significantly. With the rapid growing interest of artificial intelligence (AI), fruit production may be replaced by agricultural robots (Zhao et al., 2016). The application of agricultural robots for picking fruit and counting generally offers solutions to the expensive cost of manual labor, labor intensiveness, growing demand for food, increasing fruit quality, etc. (Sa et al., 2016).

Fruit detection is a key intelligent technological part of the development of agricultural robots for monitoring, picking fruit, and counting. Nevertheless, fruit detection is influenced by many factors such as uneven light intensity and leaf occlusions, including a situation when the target fruit exhibits the same visual appearance as its background. Additionally, the detection accuracy, inference speed, and lightweight deployment (Lawal, 2021a) are of great significance to the fruit detection model. Over the years, many fruit detection models have been proposed and have achieved good results, but most of them remain in the theoretical stage, lack practical applications, or fail to fully solve the above problems, and require further improvement. Therefore, studying a fruit detection method that can accurately detect fruit and count in complex environments, which is both fast and deployable, is of great research value.

Using You Only Look Once (YOLO) framework for fruit detection has gained a lot of attention for many years. YOLO is a single-stage target detector that has shown excellent performance for detection accuracy and speed (Lawal, 2021a; Lawal, 2021b). Fu et al. (2021) modified YOLOv3-tiny (Redmon and Farhadi, 2018) for kiwifruit detection and achieved an average precision (AP) of 90.05% and a speed of 29.4 frames per second (fps). Tian et al. (2019) improved the YOLOv3 model to detect apples at different growth stages in orchards, and the average speed of 3.4 fps for images with 3,000 × 3,000 resolution was reported. Zheng Y. Y. et al. (2019) published an AP of 88.8% and a speed of 40 fps on muskmelon detection based on YOLOv3. Gai et al. (2021) recorded an AP of 95.56% and a speed of 35.5 fps on improved YOLOv3-tiny. Lawal (2021c) and Liu et al. (2020) demonstrated that the factors of fruit detection are solvable using an improved YOLOv3. For the detection of fruits and vegetables using YOLOv4-tiny proposed by Bochkovskiy et al. (2020); Latha et al. (2022) achieved a mean AP of 51% and a speed of 55.6 fps. Meanwhile, the proposed YOLO-Oleifera by Tang et al. (2023) based on the improved YOLOv4-tiny reported 92.07% of AP, a weight size of 29 MB, and an average speed of 32.3 fps to detect each fruit image. Parico and Ahamed (2021) improved YOLOv4-tiny for real-time pear fruit detection and achieved a speed of more than 50 fps and an AP of 94.19%, but with a weight size of 22.97 MB. Yan et al. (2021) observed an AP of 86.75% and a speed of 66.7 fps using modified YOLOv5 (Jocher et al., 2022) for apple target detection. Zhang et al. (2022) incorporated a ghost network (Han et al., 2020), coordinate attention mechanism (CAM) (Hou et al., 2021), and SCYLLA-IoU (SIoU) loss (Gevorgyan, 2022) into YOLOv5s to detect a dragon fruit in the natural environment and realized an AP of 97.4% with a weight size of 11.5 MB. Qiao et al. (2022) proposed a counting method of red jujube based on the modified YOLOv5s and reported an AP of 94% and a speed of 35.5 fps using ShuffleNetv2 (Ma et al., 2018). YOLOv7 proposed by Wang et al. (2022) was reported to have surpassed other well-known object detectors including YOLOv4 and YOLOv5. Chen J. et al. (2022) enhanced YOLOv7 using a CBAM (Convolutional Block Attention Module) for citrus detection, reaching an AP of 97.29% and a speed of 14.4 fps, and the number of parameters and computation costs were reduced to 11.21 MB and 28.71 G, respectively. Zhang et al. (2022) experimented on YOLOv7 and YOLOv7-tiny for dragon fruit detection and respectively achieved an AP of 95.6% and 96.0% including a weight size of 74.9 and 12.3 MB. Nevertheless, few researchers have focused on the number of parameters and computation costs of a fruit detection model for picking and counting fruits in complex environments. Solving this big challenge is a way to realize a lightweight real-time fruit detection model that is deployable on a low-power computing device with limited memory.

Thus, this study constructed a lightweight network model based on YOLOv5s architecture to improve the detection accuracy and speed, which can be used for the real-time detection task of fruit picking robots and low-power computing device in a complex natural environment. The main contributions are summarized as follows:

(1) Establishing a new fruit dataset of dense target images under complex conditions.
(2) The network of Stem and Maxpool was adopted in the model, respectively replacing the first convolution layer and the downsample convolution layers of the original YOLOv5s network to achieve the lightweight improvement of the model. CAM was added to the original YOLOv5s network to make the model more accurate in locating and identifying dense image fruits. The AC network that involves the feature concatenation of the convolution layers with CAM was introduced for increased precision learning. The multiscale feature fusion was strengthened by replacing the C3 network in the path aggregation network (PANet) (Liu et al., 2018) with a convolution layer.
(3) Verifying the effectiveness of the improved YOLOv5s by an ablation study and comparing it with other mainstream single-stage target detection models.

The remaining part of this paper is organized as follows. The second section discusses the methods involved in the fruit dataset, improved YOLOv5s, experimental setup, and evaluation metrics. The third section explains the obtained results and discussion, and the fourth section summarizes the conclusions.

## 2 Methods

### 2.1 Fruit dataset details

**Figure 1:**
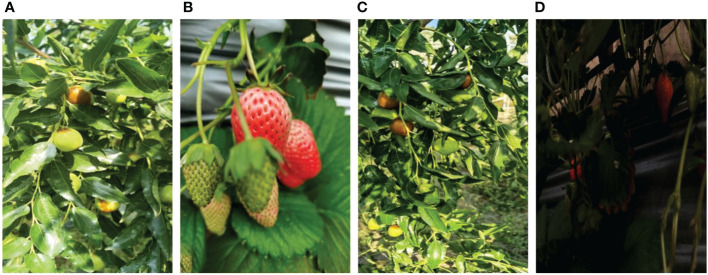
Images under different conditions: **(A)** front light, **(B)** overlapping fruits, **(C)** leaf occlusion, and **(D)** back light.

The images of strawberry (*Fragaria ananassa*) and jujube (*Ziziphus jujuba*) fruit used in this paper were respectively taken from different locations within wanghaizhuang greenhouses, Houcheng town, Jinzhong and Gaolang Red Date Picking Garden, Linxian, Luliang in Shanxi Province, China. The images were captured using digital cameras, Huawei mate30pro and mate40pro, of 3,968 × 2,976, 1,904 × 4,096, and 2,736 × 3,648 pixel resolutions, respectively, in the morning, noon, and afternoon with constantly changing distance and shooting angle. A total of 1,350 images of strawberry and 1,959 images of jujube fruit were obtained, with uneven conditions such as leaf occlusion, overlap occlusion, branch occlusion, similar visual appearance to the background image, dense targets, branch occlusion, back light, front light, side light, and other fruit natural scenes, and saved in JPG format. **Figure 1** shows some of the captured images under different conditions. These images were randomly divided into 80% for the train set, 15% for the valid set, and 5% for the test set to create 2,651, 491, and 167 images, respectively, for model training and testing paradigms as shown in **Table 1**. **Table 1** provides the dataset annotation details. The ground truth bounding boxes within each image were manually drawn using the labelImg tool without taking into account the complex condition of the image, and the annotation files saved in the YOLO text format. The YOLO text format takes target class, coordinates, height, and width. According to **Table 1**, S_Boxes_ and J_Boxes_ were the boxes created from images of strawberry and jujube fruit, respectively, having a total number of boxes of 18,496, 3,232, and 1,063 generated from 2,651 images from the train set, 491 from the valid set, and 167 from the test set, respectively. The test set, being the unseen data, was labeled in order to investigate the P–R curves’ performance, including the target fruit counting of the tested model. In addition to the dataset, recorded videos of strawberry and jujube fruits in mp4 format were provided to examine the level of robustness, real-time detection speed, and open counting of detected fruit targets.

**Table 1:** Fruit dataset annotation details.

Division	Strawberry	Jujube	Images	S_Boxes_	J_Boxes_	Boxes
Train set	1,082	1,569	2,651	7,584	10,912	18,496
Valid set	199	292	491	1,311	1,921	3,232
Test set	69	98	167	426	637	1,063
**Total**	1,350	1,959	3,309	9,321	13,470	22,791

### 2.2 Original YOLOv5s

**Figure 2:**
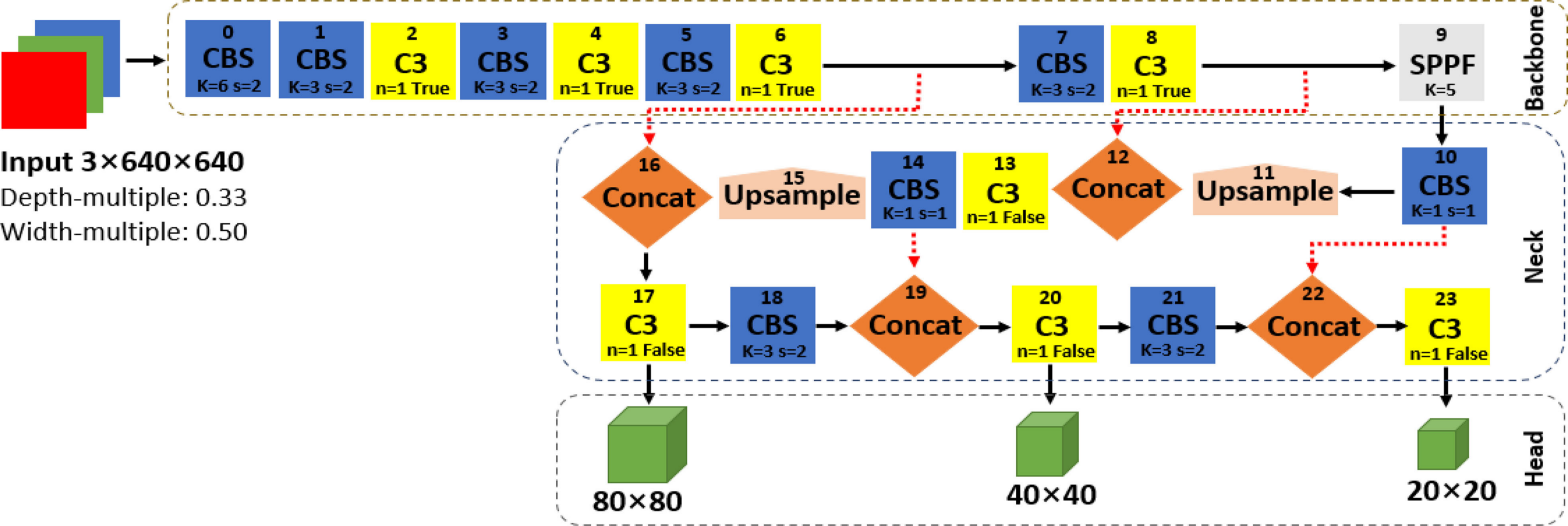
The network structure of YOLOv5s.

**Figure 3:**
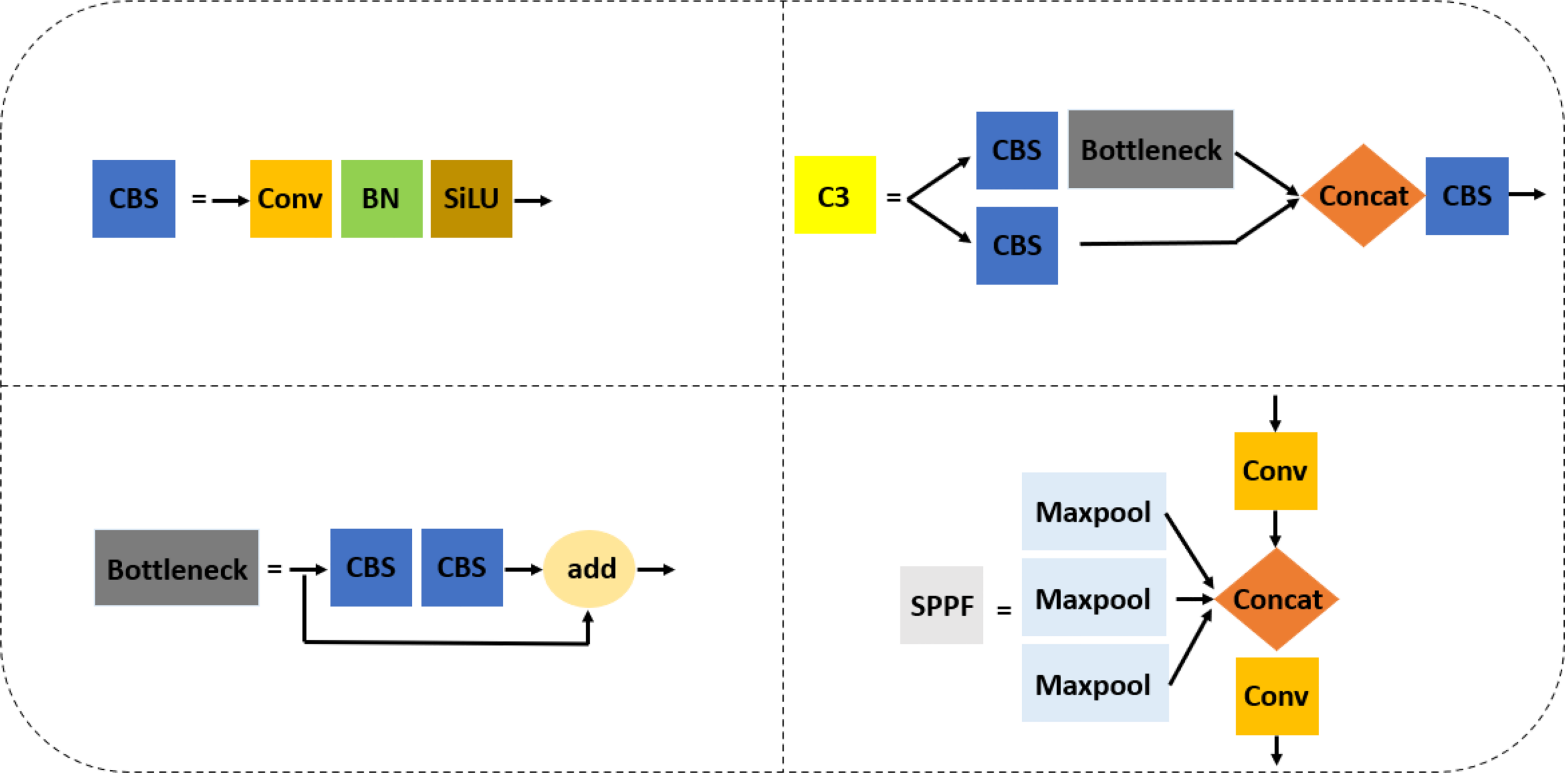
The component networks contained in the original YOLOv5s.

The original network structure of YOLOv5s shown in **Figure 2** is divided into the input, backbone network, neck network, and head network. The input integrates mosaic data augmentation, adaptive anchor, and adaptive images scaling of 0.33 depth and 0.50 width. The backbone is a convolutional neural network used to accumulate fine-grained images and generate feature maps. It contains CBS, C3, and SPPF for feature extraction as detailed in **Figure 3**. The neck part of YOLOv5s adopts the PANet structure for multiscale feature fusion. The neck network combines the feature maps collected by the backbone network and then passes the integrated feature maps to the head network, which generate predictions from the anchor boxes for object detection (Rahman et al., 2022). The head network outputs a vector with the category probability of object target, object score, and position of the bounding box surrounding the object target.

### 2.3 Improved YOLOv5s

A lightweight neural network model with high detection accuracy and speed based on the YOLOv5s network structure was proposed to support the real-time detection task of a fruit picking robot and low-power computing devices in complex natural scenes.

**Figure 4:**
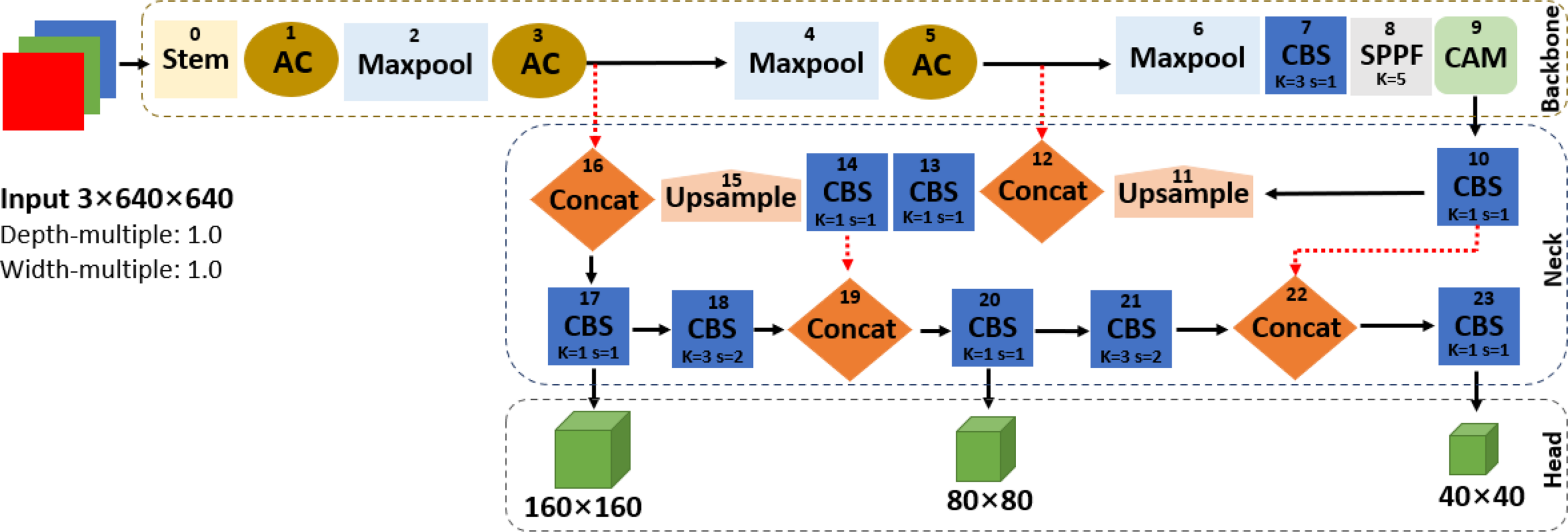
The network structure of the improved YOLOv5s.

Firstly, the adaptive image scaling of YOLOv5s was increased to 1.0 depth and 1.0 width multiples in the improved YOLOv5s network shown in **Figure 4**. This is basically to adjust the depth and width of the network to meet the needs of different scenes and improve detection accuracy, which is similar to the idea of YOLOv5l and YOLOv7-tiny. Furthermore, the adaptive anchor boxes of improved YOLOv5s were calculated using the k-means clustering algorithm to match the annotated boxes for improved fruit detection performance. The anchor box size was calculated to meet the requirements of the dataset where the best recall must be greater than 0.98, and if not, the network parameters are updated in the reverse direction.

**Figure 5:**
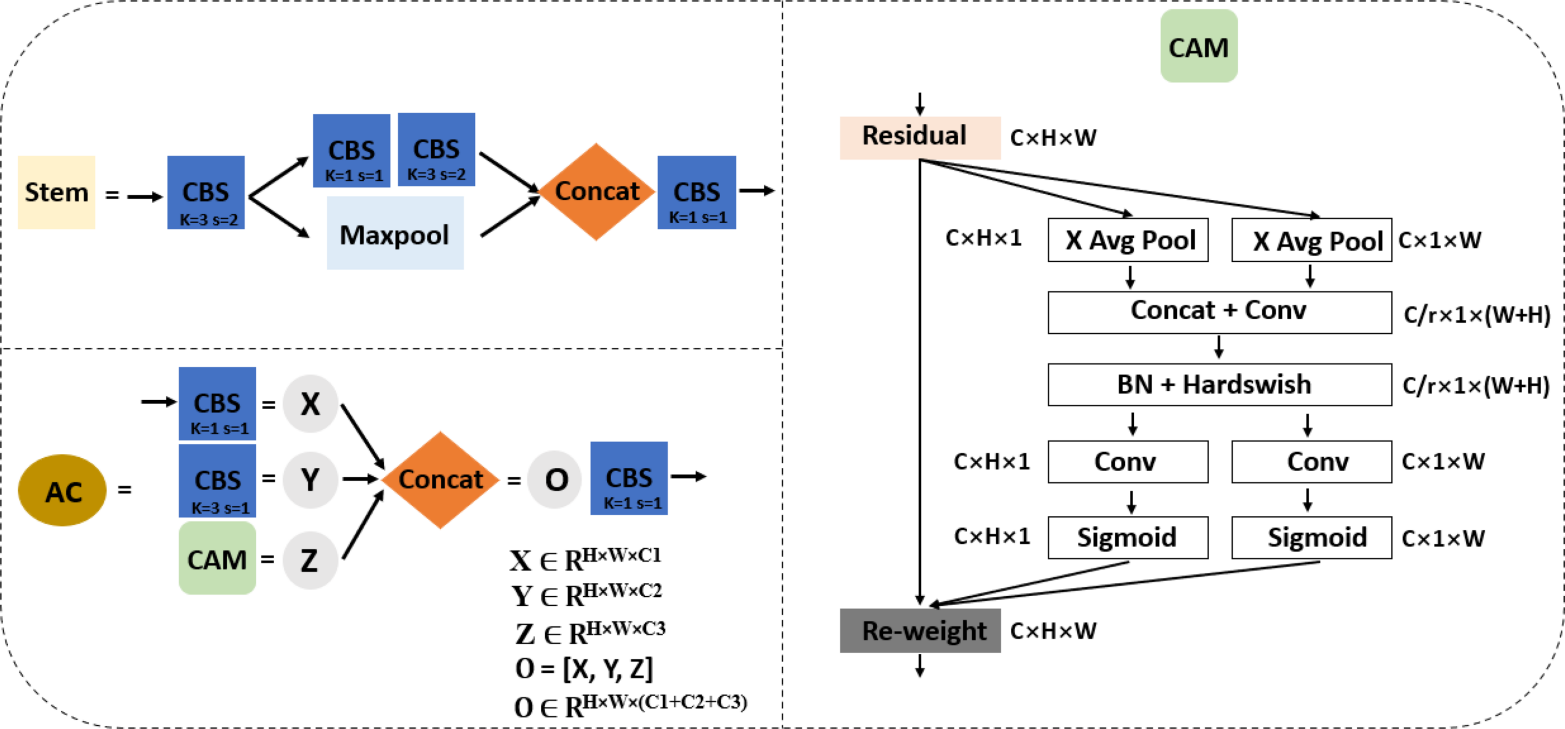
The component networks added to support the improved YOLOv5s.


Secondly, four downsampling feature maps were applied in the backbone network of the improved YOLOv5s instead of five downsampled feature maps adopted by the original YOLOv5s in order to ensure small and dense target detection. The backbone of improved YOLOv5s contains a network of Stem, AC, Maxpool, CBS, SPPF, and CAM as shown in **Figure 4**, and a component outline as shown in **Figures 3**, **5**. The lightweight Stem as the first spatial downsampling network was used to replace the first CBS of the original YOLOv5s backbone network by combining a small number of convolution kernels, which effectively reduces the computational cost while ensuring the integrity of the feature information. The AC network that concatenated 1 × 1 CBS, 3 × 3 CBS, and CAM followed by 1 × 1 CBS as depicted in **Figure 5** replaces the first, third, and fifth layers of the original YOLOv5s network. The purpose of using this network is to enhance the capacity to learn more diverse features by expanding the number of channels and, at the same time, reducing parameters and complexity. The AC network draws on the experience of selective feature connection mechanism (SFCM) (Du et al., 2019) by information sharing where the complementary features of low layers concatenate high layers. The feature concatenation is defined by **Equation (1)**, where X ∈ R^H×W×C1^ is for 1 × 1 CBS, Y ∈ R^H×W×C2^ is for 3 × 3 CBS, Z ∈ R^H×W×C3^ is for CAM, and O ∈ R^H×W× (C1+C2+C3)^ is the concatenated features of C1+C2+C3 channels, height (H), and width (W). **Figure 5** of the AC network describes the feature concatenation process stated in **Equation (1)**. The CAM proposed by Hou et al. (2021) for mobile network attention mechanism is part of the concatenation process in the AC network. With the added location information into channel attention, the CAM module can easily alleviate the loss problem of feature information of small dense objects according to Zhang et al. (2022). The imbibed Maxpool network into the improved YOLOv5s in **Figure 4** is used for spatial downsampling, which replaces the second, fourth, and sixth layers of the original YOLOv5s. The main idea is to reduce the computational cost by reducing the amount of parameters to learn and provide a faster detection speed. Additionally, the inserted networks of CBS, SPPF, and CAM respectively replaces the seventh, eighth, and ninth layers of the original backbone of YOLOv5s to foster the detection performance of improved YOLOv5s in **Figure 4**. As detailed in **Figure 3**, CBS is a convolution layer activated with SiLU (Stefan et al., 2017) after the batch normalization (BN) layer. SPPF is a feature enhancement network that helps to reduce missed target detection and enables a faster detection speed according to Jocher et al. (2022) and CAM module; the effect of enhancing representation can accurately locate and identify the dense image fruit.

(1) O=[X,Y,Z]

Thirdly, the C3 module in the neck network of the original YOLOv5s was replaced by 1 × 1 CBS and its number of networks was pruned to one on the improved YOLOv5s. The 1 × 1 CBS replaces the 13th, 17th, 20th, and 23rd layers of PANet to reduce the number of parameters and promote a faster detection speed. PANet conveys stronger localization features from the lower feature maps to the higher feature maps, which enhance the feature fusion capability of the neck network.

Finally, the head network of the improved YOLOv5s in **Figure 4** produces a feature map with dimensions of 160 × 160, 80 × 80, and 40 × 40 against the 80 × 80, 40 × 40, and 20 × 20 of the original YOLOv5s in **Figure 2**, used to detect the image targets of different sizes. This aims to improve the detection of small dense targets and speed up detection. Similar to the original YOLOv5s, the non-maximum suppression (NMS) was adopted to select the appropriate fruit targets by removing duplicate predicted boxes and complete intersection-over-union (CIoU) loss function in **Equation (2)** proposed by Zheng Z. et al. (2019) and was utilized for the convergence speed of the model network and localization accuracy with special attention to the overlap area (S), centroid distance (D), and aspect ratio (V) of the predicted box (B) and real box (B^gt^). S, D, and V are normalized from 0 and 1, and invariable on the regression scale. This measure is to enhance fruit detection performance.


(2) $$L_{\text{CIoU}}=\text{S}\left(\text{B},\;{\text{B}}^{\text{gt}}\right)+\text{D}\left(\text{B},{\text{B}}^{\text{gt}}\right)+\text{V}\left(\text{B},\;{\text{B}}^{\text{gt}}\right)$$

### 2.4 Experiment setup

The training and testing of this research work were experimented using a computer having an Ubuntu22.04LTS operating system, Core i7-12700 CPU @ 64-bit 4.90 GHz, 32 GB RAM (NVIDIA GeForce RTX 3060 GPU), python 3.9.12 and torch-1.11.0+cu113. The improved YOLOv5s including other compared models used in this paper received an input image of 640 × 640 pixels, 16 batch size, 0.937 momentum, 0.0005 weight decay, 0.2 IoU, 0.015 hue, 0.7 saturation, 0.4 lightness, 1.0 mosaic, 0.9 scale, 0.2 translate, 0.15 mix-up, and 300 epochs for training. Random initialization technique was utilized to initialize the weights for training all the models from scratch.

### 2.5 Evaluation metrics

The evaluation metrics used for fruit detection performance are precision (P), recall (R), F_1_, average precision (AP), mean average precision (mAP), speed, layers, number of parameters (params), giga floating point operations per second (GFLOPs), and weight size. The P, R, F_1_, AP, mAP, speed, params, and GFLOPs can be defined using **Equations (3)–(10)**, respectively. TP is true positive (correct detections), FN is false negative (missed detections), FP is false positive (incorrect detections), and P_(R)_ denotes that P is a function of R. F_1_ is the trade-off between P and R, AP is the P−R curve of a single class, mAP is all the AP values averaged over different classes, C is the number of classes, j is the serial number, i is the input size, k is the convolution kernel size, o is the output size, and H × W is the size of the outputted feature map. The fruit detection model tends to perform better with an increase in mAP. Speed is measured in frames per second (fps). Params is used to measure the model complexity. Layer is a network topology of the model. GFLOPs is the speed of the model based on computation costs. Size measures the model weight.


(3) $$\text{P}=\frac{\text{TP}}{\text{TP}+\text{FP}}$$

(4) $$\text{R}=\frac{\text{TP}}{\text{TP}+\text{FN}}$$

(5) $${\text{F}}_{1}=\frac{2\times \text{R}\times \text{P}}{\text{R}+\text{P}}$$

(6) $$\text{AP}={\int_{0}^{1}\text{P}}_{\left(\text{R}\right)}\text{dR}$$

(7) $$\text{mAP}=\frac{\sum_{\text{j}=1}^{\text{C}}\text{A}{\text{P}}_{j}}{\text{C}}$$

(8) Speed=frames/time

(9) params=[i×(k×k)×o]+o

(10) GFLOPs=H×W×params

## 3 Results and discussion

### 3.1 Fruit detection

**Figure 6:**
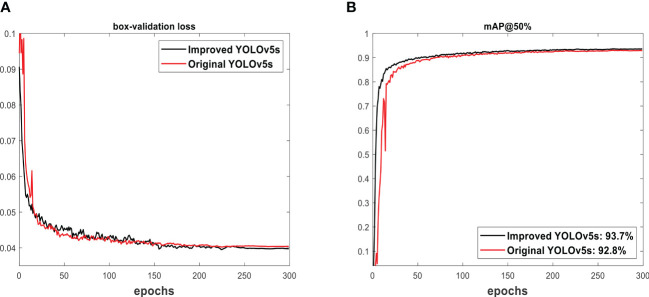
The training outcome of models: **(A)** box-validation loss and **(B)** mAP@50%.

The displayed box validation loss in **Figure 6A** measures the actual position of target fruits in an image. It shows a consistent decreasing pattern to predict the training performance of the model. The obtained box validation loss of the improved YOLOv5s is lower than that of the original YOLOv5s, confirming a deeper neural network. As the model learns, the performance improves. This decreasing box validation loss constituted an increasing mAP seen in **Figure 6B**. The 93.7% of mAP obtained from the improved YOLOv5s is higher than the 92.8% of mAP found in the original YOLOv5s model. This confirms the training superiority performance of the improved YOLOv5s over the original YOLOv5s.

**Figure 7:**
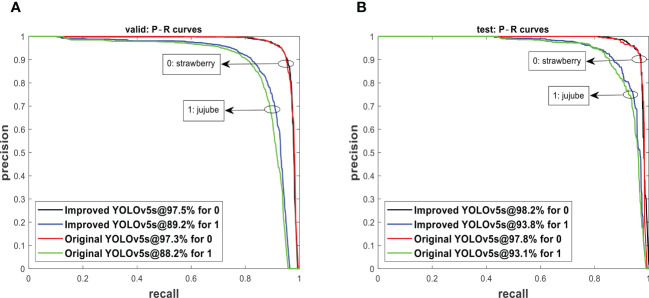
The P−R curves from **(A)** the valid set and **(B)** the test set tested on models.

**Figure 8:**
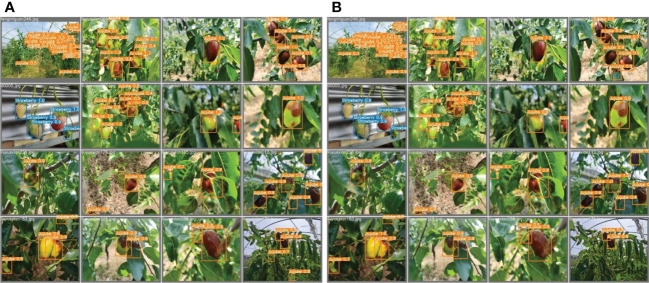
The output of images tested on **(A)** the improved YOLOv5s and **(B)** the original YOLOv5s model using the valid set.

**Figure 9:**
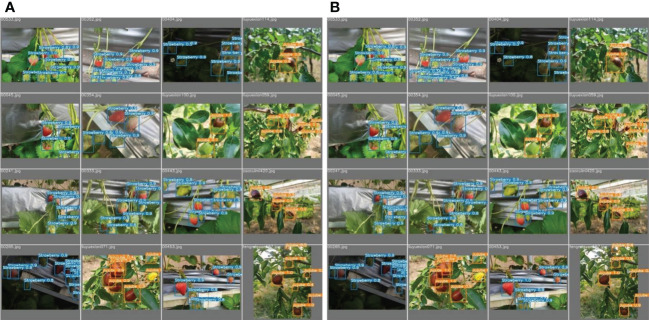
The output of images tested on **(A)** the improved YOLOv5s and **(B)** the original YOLOv5s model using the test set.

The improved YOLOv5s and original YOLOv5s were subjected to the valid set and test set using the P−R curve method as depicted in **Figure 7**. A P−R curve with a larger area under the curve (AUC) performs better. **Figure 7** shows that the AUC of the improved YOLOv5s is greater than that of the original YOLOv5s. The P−R curves under **Figure 7A** indicated that the improved YOLOv5s having 97.5% and 89.2% for strawberry and jujube targets, respectively, are higher than the original YOLOv5s having 97.3% and 88.2% for strawberry and jujube targets, respectively. At the same time, the P−R curves under **Figure 7B** show that the same trend as the improved YOLOv5s is also more than the original YOLOv5s with 0.4% and 0.7% for strawberry and jujube targets, respectively. Meanwhile, it was observed that the level of P−R curves of strawberry targets is higher than that of jujube targets as depicted in **Figure 7**. This phenomenon can be attributed to a more complex condition of jujube fruits in terms of their image background compared to strawberry fruits. For justification, the output of images tested using the valid set and test set is displayed in **Figures 8**, **9**, respectively. The predicted boxes in orange color are jujube targets while the ones in blue color are strawberry targets. A number of target fruits were detected using both the improved YOLOv5s and the original YOLOv5s model. Nevertheless, the correct detection score of fruit targets found in **Figure 8A** of the improved YOLOv5s is more than that found in **Figure 8B** of the original YOLOv5s, having higher missed detections. This is verifiable using the confusion matrix because it provides a holistic view of comparing the actual targets against the predicted targets of fruits in **Figures 8**, **9**. In the case of **Figure 9**, based on the test set, the correct detection score in **Figure 9A** of the improved YOLOv5s is 96% for strawberry and 93% for jujube targets compared to that in **Figure 9B** of the original YOLOv5s with 96% of strawberry and 91% of jujube targets using the confusion matrix. Just like **Figure 8**, the number of missed detections observed in **Figure 9B** tends to be greater than **Figure 9A**. A proof to support the presented results in **Figures 7A, B**, shows that the improved YOLOv5s outperformed the original YOLOv5s.

The obtained results that examine the overall performance of the improved YOLOv5s against the original YOLOv5s are displayed in **Table 2**. As part of the contribution to the fruit detection performance of the improved YOLOv5s, the layers, params, GFLOPs, and weight size reduce by 45.5%, 30.2%, 14.1%, and 31.3%, respectively, from the original YOLOv5s. These evaluation metrics are of high significance to both the training and testing process of models particularly with speed performance. Because the real-time and workable fruit detection model in low-power computing devices is dependent on the params and weight size according to Lawal (2021a) and Zhang et al. (2021), the average detection speed of improved YOLOv5s tested on the videos’ dataset is 10.4% higher than the original YOLOv5s. This demonstrated a faster detection speed with a reduced computation cost and a high level of robustness for fruit generalization. For the detection accuracy, the obtained P and R of the improved YOLOv5s is 91.8% and 88.6% under the valid set, and 92.8% and 92.2% under the test set, respectively, while that of the original YOLOv5s is 93.0% and 86.6% under the valid set, and 93.1% and 89.7% under the test set, respectively. With the application of **Equation (5)** for F_1_ calculations, the obtained 90.2% of valid-F_1_ tested on improved YOLOv5s is 0.5% greater than 89.7% of valid-F_1_ tested on the original YOLOv5s, and 92.5% of test-F_1_ tested on improved YOLOv5s is 1.1% more than 91.4% of test-F_1_ tested on the original YOLOv5s. However, mAP is more accurate than F_1_ because it measures P−R relationship globally using the average of all different classes. Therefore, the level of mAP as measured in the improved YOLOv5s is 0.6% for valid-mAP and 0.5% for test-mAP, more accurate than the original YOLOv5s, as indicated in **Table 2**. Thus, the obtained detection performance of the improved YOLOv5s is better than that of the original YOLOv5s.

**Table 2:** The overall detection performance between the improved YOLOv5s and the original YOLOv5s.

Model	Layers	Params ×10^6^	GFLOPs	Size (MB)	Valid F_1_%	Test F_1_%	Valid mAP%	Test mAP%	Speed fps
Original YOLOv5s	220	6.3	14.9	12.8	89.7	91.4	92.8	95.5	67
Improved YOLOv5s	122	4.4	12.8	8.8	90.2	92.5	v93.4	96.0	74

### 3.2 Fruit counting

Both the improved YOLOv5s and the original YOLOv5s were subjected to counting of target fruits using the test set and videos recorded to investigate the level of robustness. The process involves the tracking of each fruit class before counting. Under the influence of complex conditions, the models could detect the number of target fruits, as described in **Table 3**. IS_Boxes_ and IJ_Boxes_ are the detected boxes for strawberry and jujube fruits using the test set, while IS% and IJ% are respectively the percentage difference calculated between S_Boxes_ of **Table 1** and IS_Boxes_, and J_Boxes_ of **Table 1** and IJ_Boxes_. The number of detected targets using strawberry and jujube video are VS_Boxes_ and VJ_Boxes_, respectively. **Table 3** shows that the number of detected strawberry targets and jujube targets is respectively less than and higher than those in **Table 1** in terms of ground truth targets. Having less detected targets is attributed to missed detection, while more detected targets is attributed to incorrect detection. This further revealed a less complex background image of strawberry compared to jujube fruits. The improved YOLOv5s is excellent in counting 419 targets of IS_Boxes_ and 693 targets of IJ_Boxes_ compared to the original YOLOv5s at the same level of detection. Meanwhile, both models are associated with missed and incorrect detections. However, the number of missed detections observed on the tested improved YOLOv5s is 1.64 of IS% against the original YOLOv5s, which is 3.52 of IS% for strawberry targets. For jujube incorrect detections, IJ% is 8.79 on the tested improved YOLOv5s compared to 11.62 on the tested original YOLOv5s. Furthermore, the obtained results for tracking strawberry targets on video detected and counted 6,292 boxes on tested improved YOLOv5s, which is 194 more in detections than the original YOLOv5s with 6,098. In the case of jujube targets counted, the original YOLOv5s recorded 23 more incorrect detections compared to the improved YOLOv5s according to **Table 3**. Having experienced similar attributes from the test set and videos, the improved YOLOv5s is more robust for tracking and counting, making it the best candidate for fruit detection.

**Table 3:** Counting of target fruits detected using the test set and videos.

Model	IS_Boxes_	IJ_Boxes_	IS%	IJ%	VS_Boxes_	VJ_Boxes_	
Original YOLOv5s	411	711	−3.52	+11.62	6,098	9,097	
Improved YOLOv5s	419	693	−1.64	+8.79	6,292	9,074	

### 3.3 Ablation study

The ablation study presented in **Table 4** aims to investigate the performance effects of removing and replacing some features of the improved YOLOv5s detection model. The ablation study was carried out on the backbone and neck network. According to **Table 4**, using only the feature concatenation in the backbone network means without CAM as the attention mechanism. Similar to the strengthened PANet, the used feature pyramid network (FPN) by Lin et al. (2017) was improved by replacing the C3 module with 1×1 CBS. Method 1 is the improved YOLOv5s, Method 2 has the same backbone as Method 1 but with FPN, Method 3 has only feature concatenation with PANet, and Method 4 has only feature concatenation with FPN. The number of params and GFLOPs respectively observed in methods with PANet is 0.8 and 0.5 higher than methods with FPN. This is to say that the complexity and computation costs of Method 1 and Method 3 are greater than those of Method 2 and Method 4. Similarly, the methods having feature concatenation with an attention mechanism constitute a more complex network compared to feature concatenation without an attention mechanism. The level of methods’ complexity and computation costs influences the variation of detection speed shown in **Table 4**, where Method 4 > Method 3 > Method 2 > Method 1 with just 1 fps difference between them. Using accuracy, methods with attention mechanism performed better than methods without attention mechanism, and further improvement was observed in methods with PANet compared to FPN. Hence, Method 1 is 0.6%, 0.7%, and 0.8% more accurate than Method 2, Method 3, and Method 4, respectively, under valid-mAP, and 0.4%, 1%, and 1.1% more accurate than Method 2, Method 3, and Method 4, respectively, under test-mAP. The ablation study verified that Method 1, as the selected improved YOLOv5s model, performed best.

**Table 4:** Ablation study on improved YOLOv5s.

Model	Feature concatenation	Attention mechanism	Neck network	Params ×10^6^	GFLOPs	Valid mAP%	Test mAP%	Speed fps
Method 1	√	√	PANet	4.4	12.8	93.4	96.0	74
Method 2	√	√	FPN	3.6	12.3	92.8	95.6	75
Method 3	√	˟	PANet	4.2	11.5	92.7	95.0	76
Method 4	√	˟	FPN	3.4	11.0	92.6	94.9	77

### 3.4 Comparison of models

**Figure 10:**
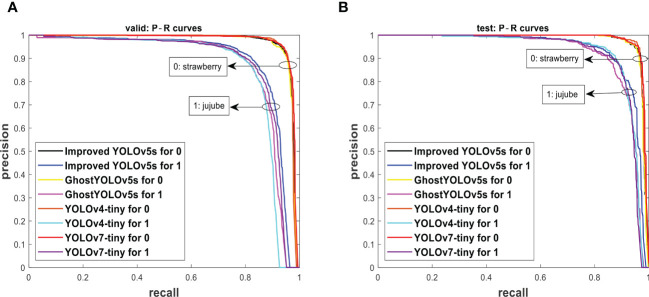
The P−R curves from **(A)** the valid set and **(B)** the test set tested on the compared models.

Using the P−R curve analysis technique, the improved YOLOv5s was compared to GhostYOLOv5s (Zhang et al., 2022), YOLOv4-tiny (Bochkovskiy et al., 2020) and YOLOv7-tiny (Wang et al., 2022) of the single-stage detection model. The same attribute of P−R curves in **Figure 7** is noted in **Figure 10**, where the P−R curves of strawberry in both the valid set and test set are greater than those of jujube fruit targets. **Figure 10A** of the valid set and **Figure 10B** of the test set indicate that the AUC of the improved YOLOv5s is greater than other models. **Table 5** reveals the overall detection performance of models to justify the displayed results in **Figure 10**. Under the accuracy performance, the improved YOLOv5s is 1%, 0.8%, and 0.3% higher than GhostYOLOv5s, YOLOv4-tiny, and YOLOv7-tiny, respectively, for valid-F_1_, and 1.2%, 0.2%, and 0.4% higher than GhostYOLOv5s, YOLOv4-tiny, and YOLOv7-tiny, respectively, for test-F_1_. For mAP, the improved YOLOv5s is 1.4%, 1.7%, and 0.5% greater than GhostY-OLOv5s, YOLOv4-tiny, and YOLOv7-tiny, respectively, for valid-mAP, and 1.0%, 0.4%, and 0.5% higher than GhostYOLOv5s, YOLOv4-tiny, and YOLOv7-tiny, respectively, for test-mAP. This demonstrates that the improved YOLOv5s is more accurate than other detection models according to **Table 5**. Meanwhile, GhostYOLOv5s has the highest number of layers at 371 compared to YOLOv4-tiny with 113, the improved YOLOv5s with 122, and YOLOv7-tiny with 208. However, GhostYOLOv5s is associated with the lowest values, 4.1 params, 9.5 GFLOPs, and 8.5 MB weight size compared to 4.8 params, 15.0 GFLOPs, and 9.6 MB weight size of YOLOv4-tiny, 6.0 params, 13.0 GFLOPs, and 12.3 MB weight size of YOLOv7-tiny, and 4.4 params, 12.8 GFLOPs, and 8.8 MB weight size of the improved YOLOv5s. Apart from the GhostYOLOv5s model, the params, GFLOPs, and weight size of the improved YOLOv5s decreases in large percentages against YOLOv4-tiny and YOLOv7-tiny. Interestingly, the obtained performance of GhostYOLOv5s was unable to obtain a faster detection speed tested on videos’ dataset, unlike other models. This outcome is linked to its large recorded layers in **Table 5**, which warrants future investigation. For other models, the detection speed of the improved YOLOv5s is equal to YOLOv7-tiny with 74 fps, higher than the 61 fps of GhostYOLOv5s and insignificantly lower than the 75 fps of YOLOv4-tiny. The detection performance in aggregation shows that the improved YOLOv5s is outstanding compared to GhostYOLOv5s, YOLOv4-tiny, and YOLOv7-tiny, including the fruit detection model proposed by Fu et al. (2021) for kiwifruits, Tian et al. (2019) for apples, Parico and Ahamed (2021) for real-time pear, Yan et al. (2021) for apples, Qiao et al. (2022) for red jujube, Chen Z. et al. (2022) for automatic estimation of apple, and Fu et al. (2022) for YOLO-Banana. For this reason, the improved YOLOv5s is lightweight with reduced computation costs, can better generalize against a fruit complex environment, and is applicable for real-time fruit detection in low-power devices.

**Table 5:** The overall detection performance comparison between models.

Model	Layers	Params ×10^6^	GFLOPs	Size (MB)	Valid F_1_%	Test F_1_%	Valid mAP%	Test mAP%	Speed fps
GhostYOLOv5s	371	4.1	9.5	8.5	89.2	91.3	92.0	95.0	61
YOLOv4-tiny	113	4.8	15.0	9.6	89.4	92.3	91.7	95.6	75
YOLOv7-tiny	208	6.0	13.0	12.3	89.9	92.1	92.9	95.5	74
Improved YOLOv5s	122	4.4	12.8	8.8	90.2	92.5	93.4	96.0	74

## 4 Conclusion

The ability to detect fruits conveniently is important for fruit picking robots. However, the fruit detection model is confronted with the challenges of a complex environment, including deployment on low-power computing devices with limited memory. For this reason, an improved YOLOv5s model with feature concatenation and attention mechanism was proposed in this paper based on YOLOv5s structure and validated using a new fruit image dataset. The improved YOLOv5s model contained the networks of Stem, AC, Maxpool, CBS, SPPF, CAM, and improved PANet to enhance the fruit detection performance. The performance demonstrated that the 122 layers, 4.4 × 10^6^ params, 12.8 GFLOPs, and 8.8 MB weight size of the improved YOLOv5s are 45.5%, 30.2%, 14.1%, and 31.3% lower than the original YOLOv5s, respectively. The obtained 93.4% of mAP tested on the valid set, 96.0% of mAP tested on the test set, and 74 fps of speed tested on videos using improved YOLOv5s is 0.6%, 0.5%, and 10.4% higher than the original YOLOv5s model, respectively. At the same time, the improved YOLOv5s is more robust for tracking and counting with less missed and incorrect detection compared to the original YOLOv5s. For the verification of effectiveness, the aggregated performance of improved YOLOv5s is outstanding compared to GhostYOLOv5s, YOLOv4-tiny, and YOLOv7-tiny models. In all, the improved YOLOv5s is lightweight with reduced computation costs, robust against complex and changeable conditions, and applicable to fruit picking robots and low-power computing devices for real-time detection. Meanwhile, decreasing the adaptive image scaling of the improved YOLOv5s model will further reduce the number of parameters and computation costs, but with a likely setback in accuracy performance. Future investigations will require improving the fruit detection performance by subjecting the proposed model to other existing neck networks and loss functions.

## Data availability statement

The raw data supporting the conclusions of this article will be made available by the authors, without undue reservation.

## Author contributions

OL was involved in setting up the software for running the experiments, preparing the image dataset, dataset annotation, data analysis, and writing and reviewing of the manuscript, while both SZ and KC were engaged in dataset annotation and data analysis. All authors contributed to the article and approved the submitted version.
